# 2D-Materials-Based Wearable Biosensor Systems

**DOI:** 10.3390/bios12110936

**Published:** 2022-10-27

**Authors:** Yi Wang, Tong Li, Yangfeng Li, Rong Yang, Guangyu Zhang

**Affiliations:** 1 School of Physics and Electronics, Hunan University, Changsha 410082, China; 2 College of Semiconductors (College of Integrated Circuits), Hunan University, Changsha 410082, China; 3 Songshan Lake Materials Laboratory, Dongguan 523808, China; 4 Beijing National Laboratory for Condensed Matter Physics and Institute of Physics, Chinese Academy of Sciences, Beijing 100190, China

**Keywords:** 2D materials, biosensors, wearable, system, power supply

## Abstract

As an evolutionary success in life science, wearable biosensor systems, which can monitor human health information and quantify vital signs in real time, have been actively studied. Research in wearable biosensor systems is mainly focused on the design of sensors with various flexible materials. Among them, 2D materials with excellent mechanical, optical, and electrical properties provide the expected characteristics to address the challenges of developing microminiaturized wearable biosensor systems. This review summarizes the recent research progresses in 2D-materials-based wearable biosensors including e-skin, contact lens sensors, and others. Then, we highlight the challenges of flexible power supply technologies for smart systems. The latest advances in biosensor systems involving wearable wristbands, diabetic patches, and smart contact lenses are also discussed. This review will enable a better understanding of the design principle of 2D biosensors, offering insights into innovative technologies for future biosensor systems toward their practical applications.

## 1. Introduction

As we reached the third year of the COVID-19 pandemic outbreak, ambitious demand has urged the revolution of life science and biotechnology in modern society. Biotechnology has emerged into a complete system including: genetic engineering [1,2], molecular biology [3], biochemistry [4], cell biology [5], embryology [6], immunology [7], and biosensing [8,9]. Among them, the biosensing technique is one of the most significant technical foundations for health testing, epidemic control, and disease diagnosis in complex situations.

Biosensing has been developed rapidly since Clark and Lyons created the use of glucose oxidase (GOD) for the electrochemical detection of glucose in 1962 [8]. It can be generally interpreted as a device that can convert a specific biological signal into a readable one. Biosensors have many ways to convert signals, mainly in optical [9,10] and electrochemical [11,12,13] approaches. Optical biosensors, as the name implies, convert biological signals into optical signals that can be read by technologies, devices, and people, thus enabling the analysis of biochemical information in biochemical reactions, biological toxins, food safety, and pharmaceuticals. Bioelectrochemical sensors can convert biological signals into readable electrical signals involving voltages, currents, frequencies, and amplitudes. To date, researchers have developed a wide range of biosensors based on metals [14], metal oxides [15], organics [16], and other materials [17,18] that can detect a wide range of human and environmental conditions [14,15,16,17,18]. The most advanced physiological biosensors are often constructed on a hard substrate with common electronic recording hardware. Hence, the monitoring sensors are usually coupled to the skin via straps/tapes with wired interfaces. Table 1 provides a comparison of different materials/techniques for the detection of breast cancer target MicroRNA(miRNA) [19,20,21,22,23,24,25,26]. From the perspective of practicality and comfort level toward final commercialization, there are still a lot of issues that need to be addressed. The problem with existing biosensors concentrates on getting them out of the lab and into the lives of users. Thus, there is a need for highly integrated wearable biosensors. Current wearable devices, typically in the form of miniaturized blocks of wireless flexible electronic/sensing components, are mainly constructed by using soft silicon [27,28] and organic polymers [29,30,31,32]. However, both materials have their drawbacks: silicon-based wearable systems struggle to maintain device performance under high strain conditions, while organic-polymers-based wearable devices have limited device performance due to their low carrier mobility.

**Figure 1:**
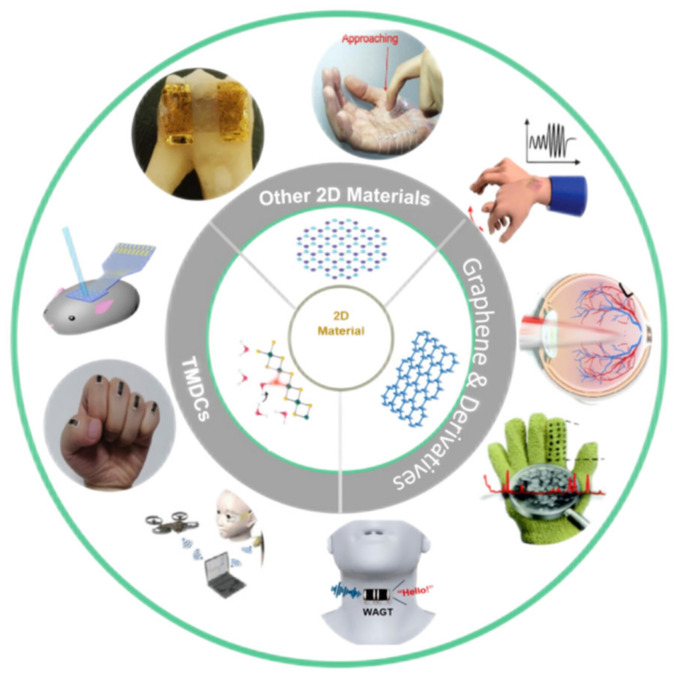
The representing sets of bio-integrated wearable sensors with 2D materials.

To address these issues, research efforts have been devoted to replacing the conventional silicon and conductive polymers with emerging two-dimensional (2D) materials. Two-dimensional materials with an atomic or molecular thickness (<1 nm) have shown several advantages such as excellent flexibility and mechanical properties, large surface area, low sheet resistance, high carrier mobility, and high biocompatibility required for the construction of wearable biosensors [33,34,35]. To date, 2D materials (e.g., graphene [36,37], transition metal dichalcogenides (TMDCs) [38,39], black phosphorus [40,41], and transition-metal carbides (MXenes) [42]) have been demonstrated with great promise in applications in biosensing technology, including electronic skins [43,44,45], contact lenses [46,47], oral sensors [48], glove sensors [49], acoustic sensors [50], and man–machine control systems [51], as summarized in Figure 1. Taking the miRNA detection as an example (Table 1), 2D materials have shown excellent properties in the field of biosensors.

This article serves as a general review of 2D-materials-based wearable biosensor systems covering their progresses, prospects, and contemporary challenges. In the following sections, we first give a brief description of the properties of 2D materials, typically graphene, TMDCs, hexagonal boron nitride (h-BN), and MXenes, followed by a discussion of varied wearable biosensor devices based on 2D materials (Section 3). Then, in Section 4, we provide the recent progress of the proof-of-concept biosensor systems involving wearable wristbands, diabetic patches, smart contact lenses, and others. Finally, we offer a summary of this review and discuss perspectives and challenges for future research and applications.

## 2. Two-Dimensional Materials

Generally, we evaluate the suitability of a material for wearable biodevices by its transmissivity, strain capacity, mechanical capacity, electrical conductivity, biocompatibility, and material size. Compared to conventional 0/1/3-dimensional materials (e.g., silicon and conductive polymers), 2D materials have better conductivity, better optical transparency, excellent mechanical flexibility, and good functionality [52]. Thus, they prevail in wearable devices as electrodes, resistors, capacitors, and field effect transistors (FETs). The properties and preparations of representative 2D materials, including graphene, TMDCs, h-BN, and MXenes, are discussed below.

### 2.1. Graphene and Reduced Graphite Oxide

As graphene was obtained by Geim et al. in 2004 [53], it has been overwhelmingly used for the fabrication of wearable systems. Its advantages include ultra-low thickness (~0.6 nm), a large theoretical specific surface area (2630 m^2^g^−1^), excellent electronic properties endowed by its carbon atom’s structure (zero band gap and high intrinsic mobility ~ 200,000 cm^2^v^−1^s^−1^), high strength (the theoretical Young’s modulus reaches 1.0 TPa) [54], high transparency, and high thermal conductivity (exceeds 5000 W/mK for monolayer graphene) [55].

Over the past decade, diverse methods have been reported for synthesizing graphene. The search for a method that can reproducibly generate high-quality monolayer graphene sheets with large surface areas and large production volumes is greatly required. Now, several physical and chemical methods have been developed to produce graphene, including the mechanical or chemical exfoliation of graphite [53], chemical vapor deposition (CVD) [56], epitaxial growth [57], reduction of Graphene Oxide (rGO) [58], and many other organic synthetic methods [59]. In addition, graphene can be modified to derive many composite materials, thus providing a more viable option for flexible biosensor fields.

### 2.2. Transition Metal Dichalcogenides (TMDCs)

TMDCs are a class of 2D semiconductor materials with important applications. They are a kind of layered compound denoted by the chemical formula of MX_2_ (M = transition metals such as Ti, V, Ta, Mo, W, and Re; X = sulfur group elements such as S, Se, and Te). Similar to graphite, TMDCs have a layered structure in which the monomolecular layers of TMDCs are stacked together by van der Waals forces. Each TMDC monolayer consists of three atomic layers, with a transition metal layer sandwiched between two sulfur layers [60]. Unlike the zero-energy gap graphene, TMDCs usually possess the semiconductor characteristic with a suitable direct band gap, which is ideal for the construction of photoelectronic devices and electronics devices with high on–off ratios. TMDCs also exhibit an ultra-sensitive response to external stimuli such as mechanical forces, light, electrical potential, molecular, and biochemical perturbations, thus demonstrating great potential for sensor applications [61].

Similar to the preparation of graphene, mechanical and chemical exfoliations [62,63] are suitable for the production of TMDCs. In addition, bottom-up growth shows superiority in the synthesis of TMDCs, especially large-scale films for practical applications. Bottom-up growth involves various methods, such as CVD [64], metal–organic chemical vapor deposition (MOCVD) [65], physical vapor transport [66], recrystallization [67], atomic layer deposition (ALD) [68], and magnetron sputtering, [69]. Recent synthesis technologies have shown that such 2D TMDC semiconductors could be available at a wafer-scale with high quality, especially for MoS_2_ [70]. Among the more than 40 species of TMDCs, MoS_2_ is the most representative one with N-type semiconducting characteristics (1.8 eV direct bandgap for monolayer MoS_2_) and chemical stability. MoS_2_ field-effect transistors (FETs) have shown high on/off ratios (>10^6^) and high mobility (217 cm^2^V^−^^1^s^−^^1^) [71]. MoS_2_ is also attractive as a potential complement for constructing flexible sensors [72,73], flexible memories [74,75], flexible organic light-emitting diode (OLED) displays [76], flexible FETs [77], etc. Moreover, these flexible FETs and circuits based on MoS_2_ have been demonstrated with high performance. As reported by Li et al., transparent MoS_2_-based transistors on four-inch flexible substrates show excellent performance with a high on/off ratio (10^10^), high current density (~35 μA μm^−1^), and high mobility (~55 cm^2^ V^−1^ s^−1^) and flexibility [70].

### 2.3. Other 2D Materials

The properties of 2D materials are closely related to their band structures. Other 2D materials such as h-BN and 2D metal MXenes, which have different energy gaps, also attract much attention.

h-BN with a band gap of 6 eV is a rare insulator in the 2D family. The layered structure of h-BN is similar to graphene, with B and N atoms bonded to each other in the same layer. In general, h-BN is obtained by mechanical stripping, CVD, liquid phase stripping, physical deposition, etc. [78]. To ensure its gas sensing capability, h-BN is usually functionalized by different modifications [79].

Metal carbides and nitrides (MXenes) are large in number and varied in category. With their excellent electrical conductivity, large surface area, and flexibility, they are widely employed in biosensing, electrocatalysis, and energy storage, occupying an important position in flexible sensing of 2D materials [80,81]. In addition, the large layer spacing of MXenes results in large resistance changes during deformation; therefore, they are often used in piezoresistive sensors such as electronic skins [82,83].

With the increasing development of various 2D materials, scientists’ research goals are no longer limited to 2D planes, but turn to heterostructures, such as graphene/MoS_2_, MoS_2_/WSe_2_, and Graphene/BN [84]. This kind of heterostructure stacks 2D atomic crystal materials with different electrical and optical properties together to form double-layer or even multilayer artificial materials maintained by van der Waals forces. It is an exotic degree of freedom to enable various fascinating new phenomena in 2D van der Waals heterostructures with adjustable energy band arrangement structures [84]. The nearly infinite possibilities make the 2D van der Waals heterostructure even more important than the 2D materials themselves in device applications.

## 3. Wearable Biosensors Based on 2D Materials

The ultra-low thickness and excellent physical properties have made 2D materials more attractive in the research of flexible wearable devices. This section describes the fundamental concepts, working mechanisms, and performance of various 2D biosensors.

### 3.1. E-Skins

Electrical skin (e-skin), aiming to visualize the information received by human skin into signals, is one of the significant members of wearable sensors. Numerous types of e-skins were investigated and introduced into services, such as health detection, external environment monitoring, human–computer interaction, and robot control. One important function of human skin is haptics, or its ability to sense pressure, which can sense down to 2 kPa. Hence, the fundamental function of an e-skin is to mimic this pressure sensitivity. To achieve this, various methods are exploited to fabricate e-skin with different modes, including resistive [85], capacitive [86], optical [87], and piezoelectric methods [88,89]. Table 2 summarizes the performance of these four types of e-skin.

**Figure 2:**
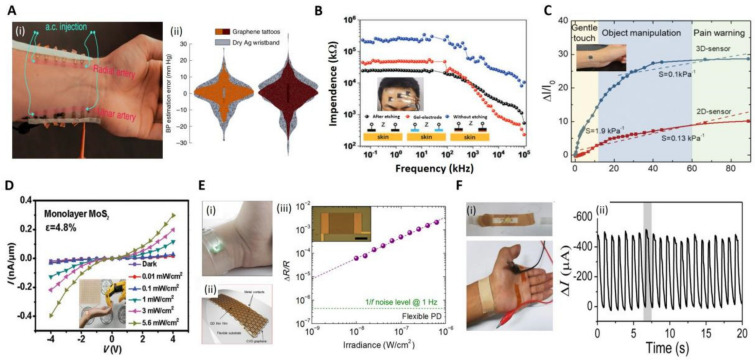
Several e−skins based on 2D materials. (**A**): (i) Photo of the graphene bioimpedance tattoos attached to human skin. (ii) Comparison of statistical violin plots of graphene Z−BP versus commercial dry silver wristbands (diastolic (left) and systolic (right)) [97]. Copyright 2022, Springer Nature. (**B**): Impedance difference between LSG/PU e−skin with and without etched PU and commercial gel electrodes during measurement (the illustration shows the optical image when worn on the user’s head) [98]. Copyright 2022, Wiley-VCH. (**C**): Relative current change versus applied pressure for the pressure sensors based on 3D microelectrodes and 2D flat electrodes, where S represents the sensitivity of the pressure sensor; inset shows a photo of the sensor attached to the skin [86]. Copyright 2019, Wiley−VCH. (**D**): Current versus source−drain voltage on a single layer of MoS_2_; illustration of this elastomeric substrate attached to a human wrist for lighting detection and human–machine interaction [90]. Copyright 2022, Wiley-VCH. (**E**): (i) Physical diagram of reflection mode photodetector. (ii) Schematic illustration of the assembly of graphene and QDs on a flexible substrate. (iii) Photo-induced resistance change (ΔR/R) with respect to irradiance at 633 nm. [87]. Copyright 2019, American Association for the Advancement of Science. (**F**): (i) Photograph of the measurement for wrist pulses. (ii) 20 s real-time record of wrist pulses [91]. Copyright 2017, American Chemical Society.

The first type of e-skin is a resistive electronic skin sensor, which can detect pressure by the change in resistance (R) when deformation occurs on its component materials. It can measure resistance through a constant power supply and output electrical signals in real time with high sensitivity, low cost, and high thermal stability. However, it suffers from drift and hysteresis. As one of the most conductive materials (only 10^−6^ Ω•m), graphene is a suitable material for resistors [92,93,94,95,96]. Kireev et al. developed a wearable continuous blood pressure (BP) monitoring platform by using graphene electronic tattoos as human bioelectronic interfaces (Figure 2A). This device provided a noninvasive, continuous recording of blood pressure with an accuracy of 0.2 ± 4.5 mmHg for diastolic and 0.2 ± 5.8 mmHg for systolic measurements, which is comparable to Class A devices now available for sale. They continue improving its accuracy, eliminating noise and motion artifacts through making it learn regression models. These graphene electronic tattoos can monitor arterial BP for >300 min, a period ten-fold longer than reported in previous studies [97]. Figure 2B shows a combined laser scribed graphene (LSG) technology by Qiao et al. with polyurethane (PU) to create an e-skin with large measurement range (with a strain of 60%), high sensitivity (gauge factors = (ΔR/R_0_)/(ΔL/L_0_) ≈ 40), and high linearity range (with a strain of 60%). This kind of e-skin has shown greatly reduced impedance in detecting electrophysiological signals and, thus, has been used to monitor electrocardiogram (ECG), electroencephalogram (EEG) and electro-ocular signals [98]. Yao et al. constructed a deformed tattoo with a cracked graphene structure by laser scribing. The e-skin is endowed with contactless temperature-sensing capability by the excellent light and thermal sensing properties of graphene. Moreover, the other properties, e.g., ultrafast responsiveness (6.7 ms) and resilience (13.4 ms), a broad pressure sensing range (2.5 Pa–1.1 MPa), a high sensitivity (2.14 kPa^−1^), and robust cyclability (2000), make the e-skin more similar to human skin [85].

The second type of e-skin is a capacitive sensor, which can measure pressure by changing the distance between the upper and lower plates under pressure, then the capacitance is changed and recorded by an electrical output signal [99,100,101]. The advantages of capacitive sensors are low-power consumption, high static stability, and high sensitivity. However, they are more susceptible to external interference and often costly. Figure 2C shows a flexible pressure sensor with ultrathin and ultralight features fabricated by Han et al. The transfer technology was employed to fabricate the electrode and sensing layers with different microstructures. The sensitivity of the sensor was enhanced by the 3D-microstructured electrodes and the irregular rough micropatterned sensing layer. Therefore, the pressure sensor exhibited striking characteristics, including high pressure sensitivity (6.13 kPa^−1^), wide detection range (from 20 Pa to 90 kPa), and low operating voltage (0.1 V). As a result, the sensor almost covers the entire sensing range of human skin with the capability of detecting a variety of pressure stimuli, such as radial pulse, human movement, and the touch of very small objects [86].

The third type of e-skin is the optical sensor, which converts pressure signals into electrical signals through an optical technique [87,90,102,103]. Among all the types of sensors, it is superior in its sensitivity, static terms, linearity, and resistance to drift; however, its instability in the worn situation, high-cost manufacturing, and energy intensity limit their applicability in small, simple, and low-power e-skin devices. Zhang et al. used a multilayer MoS_2_ stacking strategy to fabricate an optical sensor (Figure 2D) that could be attached to human skin to control a robotic hand for human–computer interaction at a 16% strain level (Figure 2D) [104]. Polat et al. fabricated a photodetector (PD) with semiconductor quantum-dot-sensitized graphene (GQD) (Figure 2E). It can be integrated as a module in a flexible printed circuit board (PCB), linked Bluetooth, and near-field-communication (NFC) module. Applications of the device include a pulse detector, a health patch for mobile phone screens, and a wireless UV detection sensing patch [87].

The fourth type of e-skin is a piezoelectric sensor, which converts the mechanical energy of pressure into electrical energy and outputs an electrical signal [91,104,105,106]. It is advantageous in fast response, energy efficiency, and accuracy, but is disadvantageous in its complexity, high-cost manufacturing, and susceptibility to crosstalk, which makes it difficult for commercialization. However, great efforts have been made to improve its reliability through artificial intelligence (AI). As shown in Figure 2F, Chen et al. proposed a piezoelectric pressure sensor with nanowires/graphene heterostructures capable of measuring static pressure with a sensitivity of up to 9.4 × 10^−^^3^ kPa^−^^1^ and a fast response time down to 5–7 ms (the sensitivity is expressed as a ratio of current variation over its initial value) [91]. This demonstration of pressure sensors shows a great potential in the applications of e-skin and wearable devices.

Another important function of human skin is excretion. Containing various physiological indicators such as electrolytes, amino acids, cortisol, glucose, lactic acid, and excretion serves as a reflection of human diseases. Bariya et al. created a glove to physically collect human sweat to detect physiological indicators [107]. Cui et al. fabricated a β-cyclodextrin (β-CD)-functionalized graphene-based e-skin to detect K^+^ and pH in sweat by using a β-CD-functionalized printed flexible graphene electrode as the pH sensing electrode and a flexible silver electrode as the reference electrode [108]. Liao et al. fabricated a multiplexed sweat analysis e-skin based on a laser-induced 3D porous graphene (LIG) electrode on polyimide (PI) film. For the Na^+^- and K^+^-selective sensors, the sensing electrodes were modified by poly (3,4-ethylene dioxythiophene): polystyrene sulfonate (PEDOT: PSS), which is an excellent ion-to-electron transducer. For the pH sensor, an H ion-selective electrode was modified by polyaniline (PANI) using cyclic voltammetry. The LIG-based sensors showed good performance, with sensitivities of 51.5 mV/decade (pH), 45.4 mV/decade (Na^+^), and 43.3 mV/decade (K^+^), and the sensing performance was well-maintained under bent states. Good reproducibility, stability, and selectivity were also observed [109].

Having achieved the detection of physiological indicators on the body surface, researchers have paid more attention to the environmental information in contact with the body surface. A new strategy has been used to achieve the detection of NO_2_ in the environment by exposing the edge sites of MoS_2_ and WS_2_ to construct heterogeneous junctions [110]. Lee et al. constructed a laboratory jacket by assembling graphene and MXene layer by layer to achieve the detection of NH_3_. The graphene-hybrid-based fibers have shown good mechanical flexibility (bending for 2000 cycles, resistance fluctuation ±0.2%) and a high sensitivity of 6.77% (∆R/R_0_ = 6.77%) to 50 ppm NH_3_ at room temperature [111].

Significant progress in the development of e-skin has been achieved in recent years; however, as single-function e-skin products have reached a summit, there is a significant opportunity to develop multifunctional e-skins, on which different sensors, communication modules, and power supply systems are integrated.

### 3.2. Contact Lens

As one of the vital organs of human beings, the eyes can reflect numerous physiological indicators through physical and chemical information. For example, intraocular pressure is the main diagnostic indicator of glaucoma, and the temperature of the eye reflects the level of fatigue or post-operative inflammation. The chemical information contained in tears can be seen as an indicator of the chemical information in blood. As many people show reluctance and fear of invasive blood collection, contact lenses, as a wearable and continuous diagnostic biosensing device, have become a more acceptable substitution. The electrolytes in tears such as K^+^, Na^+^, Ca^2+^, Mg^2+^, and Cl^−^ can be converted into blood electrolyte levels. For example, urea reflects the health of kidney function, glucose is present as an indicator of diabetes, various proteins are associated with diseases such as inflammation, AIDS, and cancer, and cortisol reflects the level of stress as some studies have shown a relationship between cortisol and diabetes [112,113,114,115].

**Figure 3:**
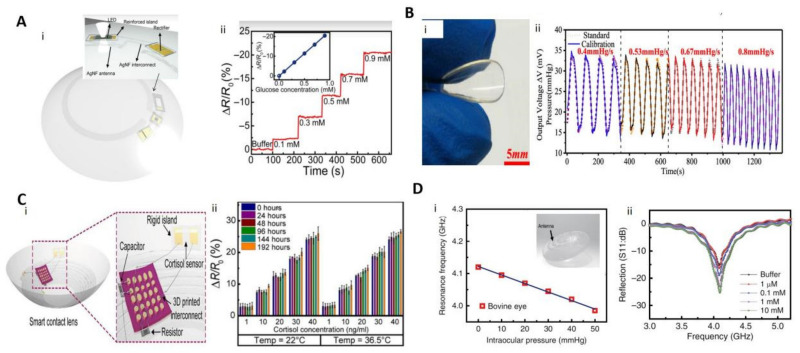
Several contact lens sensors based on 2D materials. (**A**): (i) Glucose contact lens sensor and schematic diagram of the structure. (ii) Variation in the resistance of the sensor in different concentrations of glucose [117]. Copyright 2018, American Association for the Advancement of Science. (**B**): (i) Optical photograph of the IOP contact lens sensor. (ii) Comparison between the calibrated IOP and the standard IOP at different speeds [118]. Copyright 2020, American Chemical Society. (**C**): (i) Cortisol contact lens sensor and schematic diagram of the structure. (ii) Variation in the resistance of the sensor at different temperature and time states [119]. Copyright 2020, American Association for the Advancement of Science. (**D**): (i) Frequency response of the intraocular pressure sensor on the bovine eye from 5 mm Hg to 50 mmHg. Illustrations show schematic diagram of the structure of the composite contact lens sensor. (ii) Wireless monitoring of glucose concentrations from 1 μM to 10 mM [47]. Copyright 2017, Springer Nature.

Excellent transparency, flexibility, and biocompatibility have made 2D materials a viable option for contact lens sensors. Similar to their roles in e-skin fabrication, 2D materials generally serve as resistors, capacitors, electrodes, and key devices for sensing in contact lens sensors. For example, researchers have used graphene-coated contact lenses to reduce electromagnetic field interference and induce dehydration protection [116]. Park et al. showed a smart contact lens based on functionalized graphene as a glucose sensor. They used silver nanowires as a circuit, silicon-based chips as diodes, graphene functionalized by glucose oxidase as electrodes, and took the LED as an indicator (Figure 3A). When wirelessly powered via a resonant coil, the power is transmitted to the contact lens sensor via the antenna. This power supply activates the LED and the sensor. The LED switches off when the glucose concentration in tears exceeds the normal level [117]. Xu et al. used the piezoresistive properties of graphene to create a graphene piezoresistive contact lens that can measure intraocular pressure (IOP) to prevent glaucoma (Figure 3B), with an average sensor sensitivity of 150 μV/mmHg and 85% transparency [118]. The few-layer graphene Wheatstone bridge consisting of two strain gauges and two compensating resistors was designed to improve the sensitivity and accuracy of IOP measurement. Testing results on a silicone eyeball in Figure 3B indicated that the output voltage of the sensor is proportional to the IOP fluctuation.

Despite various approaches to immunoassay and chromatography for monitoring cortisol concentrations, all the conventional methods require bulky external equipment, which limits their use as mobile healthcare systems. As shown in Figure 3C, Ku et al. developed a graphene field-effect-transistor-based smart contact lens for real-time detection of the cortisol concentration in tears. The graphene FET where the graphene channel is selectively functionalized with cortisol monoclonal antibody can be used as a cortisol sensor. In addition, integrated with this cortisol sensor with transparent antennas and wireless communication circuits, it can be operated remotely by a smartphone without obstructing the wearer’s view. They used NFC technology for communication and power supply and detected the cortisol secreted in tears by reading the resistance of the cortisol sensor with a stability up to 192 h at 22–36.5 °C. The detection limit of 10 pg/mL meets the normal range of human secretion [119]. There is also a desire to integrate more functionality in contact lens sensors. In Figure 3D, researchers used graphene as a channel material and source–drain FET for wireless detection of glucose, as well as graphene-Ag nanowires acting as capacitors to detect IOP [47].

Contact lens sensors have not seen innovative research for a long time, due to the limited area they can be used in, resulting in a single function, low energy transfer efficiency, and low communication data load. With advances in energy conversion/storage technology, the future of contact lens sensors may be a combination of highly integrated sensor arrays and data processing via AI algorithms in the cloud, resulting in a better user experience.

### 3.3. Other Types of Wearable Sensors

Although much of the current research on wearable sensors is focused on e-skins and contact lens sensors, other types of wearable sensors for different body organs still provide high research value.

As one of the most complex environments in the human body, the mouth contains many electrolytes, microorganisms, enzymes, proteins, gases, DNA, RNA, etc., which have high monitoring value and clinical diagnostic significance. The gases exhaled from the mouth also reflect health indicators. Gases from the human body contain components such as CO, bacteria, and volatile organic compounds (VOCs), which have clinical diagnostic significance for diabetes, cancer, gastritis, etc. Hou et al. proposed a novel humidity sensor functioning by a borophene–MoS2 heterostructure through controlled ultrasonication. The sensitivity was significantly enhanced to as high as 15,500% at a relative humidity of 97%, which is more than 90 or 70 times higher than that of a single borophene or MoS_2_ [120]. Liu et al. produced an electronic nose by constructing functionalized reduced graphene oxide to detect four cancer-related marker compounds (ethanol, 2-ethylhexanol, nonanal, and ethylbenzene) at 25 ppm with a linear response. Saliva correlates with blood and can be used to detect inflammation in various diseases [121].

**Figure 4:**
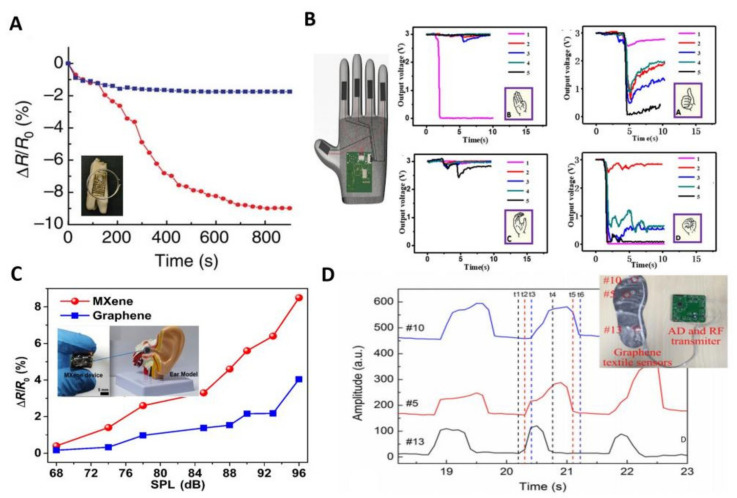
Several other wearable sensors based on 2D materials. (**A**): Percentage change in graphene resistance over time after exposure of the dental patch sensor to approximately 100 H. pylori cells in human saliva (red line). The response to a “blank” saliva solution is shown as a blue line [48]. Copyright 2012, Springer Nature. (**B**): Glove sensor for different schematics and the voltage output for different gestures [49]. Copyright 2021, Springer Nature. (**C**): Cochlear sensor resistance change with decibels; inset shows the MXene cochlear sensor [124]. Copyright 2021, Multidisciplinary Digital Publishing Institute. (**D**): Photograph of the foot sensor and comparison of the forces applied to each part(#5#10#13 in the illustration in the upper right corner correspond to the pressure sensors at different locations on the bottom of the three feet respectively.) [126]. Copyright 2017, Multidisciplinary Digital Publishing Institute.

In Figure 4A, Mannoor et al. introduced a novel approach to produce wireless graphene nanosensors onto biomaterials via silk bioresorption. Graphene nanosensors are first printed onto water-soluble silk thin-film substrates, then contacted by interdigitated electrodes. Finally, the graphene/electrode/silk hybrid structure is transferred to biomaterials such as tooth enamel or tissue. The resulting device architecture is capable of extremely sensitive chemical and biological sensing, with detection limits down to a single bacterium, while also wirelessly achieving remote powering and readout. The research is considered an important milestone for oral health sensors [48].

Human hands are used most frequently to deliver signals and information. The role of gloves in wearable devices serves to detect pressure and environmental information and transmit sign language. Li et al. integrated a batch of surface-enhanced Raman scattering (SERS) arrays composed of Ag/MoS_2_ particles on flame-retardant gloves through screen printing technology, which can detect multiple polycyclic aromatic hydrocarbons (PAHs) simultaneously [122]. Yuan et al. developed a low-cost graphene gesture sensor, which, combined with a MATLAB artificial network, can achieve accurate recognition of 26 English letters (Figure 4B) [49].

According to statistics, more than 1.57 billion people worldwide suffered from deafness by 2019 and the number might reach up to 2.45 billion by 2050 [123]. The intervention of cochlear implants and speech recognition sensors would be an effective alleviation. Li et al. reported an artificial eardrum using an acoustic sensor based on 2D MXene (Ti_3_C_2_T_x_), which can enable a two-stage amplification of pressure and acoustic sensing, thus mimicking the function of a human eardrum for realizing voice detection and recognition. As shown in Figure 4C, the MXene artificial eardrum shows an extremely high sensitivity of 62 kPa^−1^ and a very low detection limit of 0.1 Pa [124]. Later, Wang et al. designed a graphene throat patch that is capable of recording deformation resistance through weak vibrations even when no sound is emitted subsequently through AI analysis of the signal [125].

The foot is the end of the movement chain in the process of human movement. Many physical parameters can be obtained from plantar pressure distribution analysis. Figure 4D shows a pressure sensor with a graphene textile that was synthesized by reduction of graphene oxide with the help of vacuum filtration. Here, the piezoelectric properties of graphene were used to prepare a textile-embedded insole based on LSG to detect the pressure distribution under the foot by piezoelectric resistance [126].

## 4. Integrated Wearable Biosensor Systems

The 2D-based biosensors described above are focused on device function rather than system performance. The most advanced noninvasive physiological monitoring systems desire multifunctional and highly integrated sensors, which require more interdisciplinary collaboration rather than the creation of a single-functional sensor. An ideal sensor system consists of a power supply module, a communication module, a computational storage module, a sensing module, and even a drug delivery system (therapeutic module). In this section, we present some of the scientific results that have guided the 2D-based wearable biosensor systems, as well as the components needed to build a complete wearable system.

### 4.1. Two-Dimensional-Based Wearable Biosensor Systems

A high degree of integration is the way forward for the commercial operation of wearable flexible sensors. A popular wearable system must be self-contained. However, due to the multidisciplinary intersection and integration techniques in 2D materials, only a few works can achieve a fully integrated wearable biosensor system.

One representative example is a fully integrated wristband sweat sensor designed by Gao et al. They used commercially available silicon-based integrated circuit technology (more than ten chips) and five functional sensors integrated on a flexible substrate. This wearable device can communicate in real time to a mobile phone terminal or the cloud and compensate for the signal, as well as provide measurement and storage for transmission, with great potential for physiological clinical research and commercialization [127].

**Figure 5:**
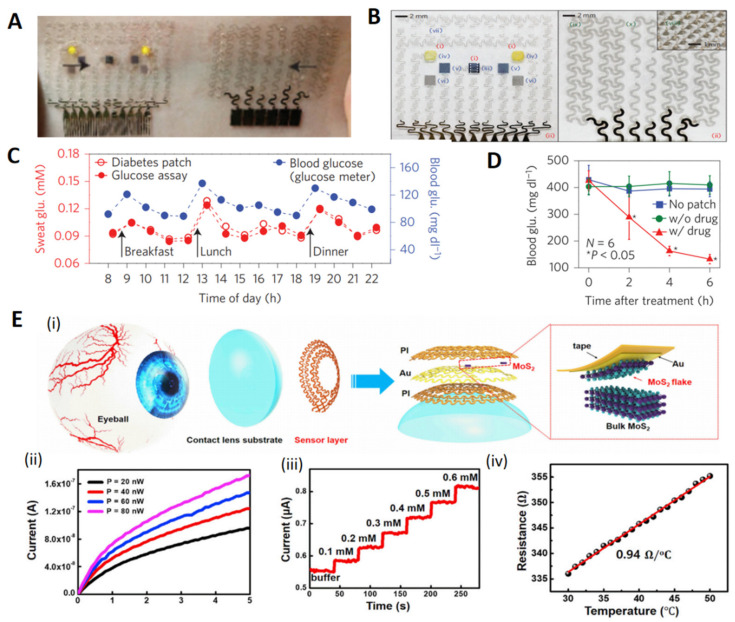
Several highly integrated wearable sensors based on 2D materials. (**A**): Optical camera images of the diabetes patch laminated on human skin. (**B**): Schematic diagram of a GP-hybrid electrochemical unit consisting of electrochemically active and soft functional material (xi), gold-doped graphene (xii), and serpentine gold mesh (xiii) from top to bottom. (**C**): One-day monitoring of human sweat and blood glucose concentrations in human sweat and blood. (**D**): Comparison of blood glucose concentrations in db/db mice in the treatment group (with drug) and control group (without patch and without drug) when used on diabetic mice [128]. Copyright 2018, Springer Nature. (**E**): (i) Schematic diagram of the different layers of the smart contact lens structure attached to the eye. The dashed area highlights the gold-mediated mechanical peeling of a single layer of MoS_2_. (ii) Leakage source characteristics of the photodetector at different light intensities. (iii) Time vs. current curves based on changes in glucose levels. (iv) Resistance versus strain of the temperature sensor at different temperatures [129]. Copyright 2020, Elsevier.

Lee et al. showed that Au-doped graphene combined with a gold mesh (Figure 5A) has improved electrochemical activity compared to bare graphene, thus sufficient to form a wearable patch for sweat-based diabetes monitoring and feedback therapy. The stretchable device features a serpentine bilayer of gold mesh and gold-doped graphene that forms an efficient electrochemical interface for the stable transfer of electrical signals. As shown in Figure 5B, the patch consists of a heater, and temperature, humidity, glucose, and pH sensors, together with polymeric microneedles that can be thermally activated to deliver drugs transcutaneously. As shown in Figure 5C,D, the patch can be thermally actuated to deliver Metformin and reduce blood glucose levels in diabetic mice [128].

Contact lens sensors, as attached to the most vulnerable part of the body exposed to air, should not only have high light transmission and low fogging, but also be comfortable and healthy. Its development highly requires the integration of technology and material technology. Guo et al. demonstrated a facile approach to fabricating multifunctional ultrathin MoS_2_ transistors and an Au-wire-based sensor system (Figure 5E). It could be directly incorporated onto soft contact lenses using an easy assembly method that leaves sensors exposed to the tear fluid, providing high detection sensitivity. The thin donut serpentine mesh architecture ensures strong mechanical robustness, high stretchability, and eye comfort while maintaining high transparency. In vitro studies have shown that this system is fully biocompatible and nontoxic to human cells [129].

The need for smarter wearable devices has emerged as an effective solution, which offers the possibility of AI. Machine learning technology can learn patterns and performance between samples of text, images, sound, etc., through a nonstop repetitive process, and this technology is used in wearable technology mainly to eliminate distractions caused by the environment and movement. Lu et al. devised a nanofriction power generation technique that supplies electricity while allowing lip reading through electrical signal output. The highlight of this study is the use of deep learning to propose an expanded recurrent neural network (expanded RNN) model based on a prototype learning approach to recognize lip variations. For each classification model, a prototype is learned in the depth feature, reducing the number of samples and improving the accuracy of recognition [130].

### 4.2. Power Supply for Biosensor System

A significant part of the current research on wearable systems requires external power supply systems to carry out, which include computers, electrochemical workstations, and multimeters. Nowadays, most of the commercial wearable devices use traditional batteries such as lithium batteries and button cells, which limit the miniaturization and application of devices. Hence, efforts have been made on the power supply. Thus far, wearable systems can be expected to be powered by supercapacitors, solar cells, bioelectricity technology, physical hair technology, NFC technology, etc.

Flexible self-healing supercapacitors, a competitive power supply system with high energy density, high charging and discharging efficiency, and excellent mechanical flexibility, meet substantially all the requirements for powering wearable sensing devices. Vu et al. demonstrated a self-healing flexible supercapacitor based on a conductive composite electrode composed of polyurethane and carbon black (PU/CB) using a sandwich structure that provided excellent electrical performance and mechanical flexibility. The device has an electrical energy density of 5.8 μWh/cm^2^ at 1 mA/cm^2^ and 91% capacity retention during 10,000 charge/discharge cycles after breaking/healing [131]. Two-dimensional materials also emerge in this area. Kumar et al. used graphene as a printing ink combined with 3D technology to produce a flexible supercapacitor without any additives [132].

**Figure 6:**
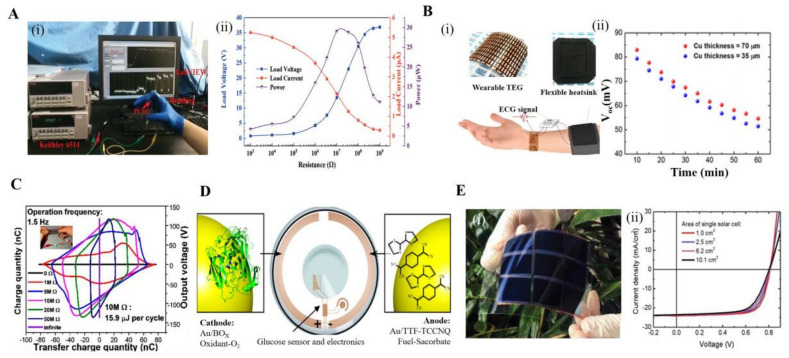
Several powered devices for wearable sensors based on 2D materials. (**A**): (i) Electrical signals generated by flexible piezoelectric nanogenerators when bent. (ii) The output voltage, current, and instantaneous power outputs dependence on different load resistance ranges [133]. Copyright 2021, Wiley-VCH. (**B**): (i) General schematic diagram of a thermogenerator self-powered wearable ECG system. (ii) System open-circuit voltage versus operating time variation [134]. Copyright 2018, American Chemical Society. (**C**): V–Q curves of a TENG under different loads. Illustration is photograph of using the integrated KP-SC (6 units; 3 devices in series) to light up a single commercial green LED under cycling stretching movement [135]. Copyright 2016, American Chemical Society. (**D**): Working of a glucose biofuel cell contact lens sensor, schematic diagram [136]. Copyright 2013, American Chemical Society. (**E**): (i) Optical image of an organic photovoltaic flexible assembly module. (ii) J–V curves of flexible photovoltaics of different areas [137]. Copyright 2021, Wiley-VCH.

Physical power generation techniques generally exploit the piezoelectric and Seebeck effects to power a thermodynamic generator by using ambient-skin-temperature differences. Lu et al. designed a flexible piezoelectric nanogenerator through 3D nano-BCZT@Ag heterostructures. The device shown in Figure 6A can be powered by human walking and can deliver 5.85 μA of current (38.6 V) [133]. Triboelectric nanogenerators (TENGs) are used to power devices by converting friction into electrical energy. As shown in Figure 6B, Kim et al. showed us a wearable ECG system based on a wearable thermoelectric generator (w-TEG) that can provide more than 13 μW/cm^2^ of power for more than 22 h through temperature differences [134]. In Figure 6C, Guo et al. presented a prototype of an all-in-one shape-adaptive self-charging power unit that can be used for scavenging random body motion energy under complex mechanical deformations. A kirigami paper-based supercapacitor (KP-SC) was designed to work as the flexible energy storage device (stretchability up to 215%). A stretchable and shape-adaptive silicone rubber triboelectric nanogenerator (SR-TENG) was utilized as a flexible energy harvesting device. By combining them with a rectifier, a stretchable, twistable, and bendable self-charging power package was achieved for sustainably driving wearable electronics. This work provides a potential platform for flexible self-powered systems [135].

Bioelectric conversion technologies are generally generated through redox reactions of electrolytes, enzymes, proteins, and other substances in body fluids. As shown in Figure 6D, Falk et al. constructed a self-powered glucose-sensing contact lens using ascorbate and oxygen from human tear fluid as the fuel and oxidant, able to deliver a stable current for up to 6 h [136].

Organic solar cells (OSCs) have attracted a lot of attention as a clean energy source due to their low cost, light weight, and flexibility. Figure 6E presents the large-area non-fullerene organic solar cells and modules based on a flexible low-work function composite electrode (Ag grid/AgNWs:PEI-Zn). Large power conversion efficiencies (PCEs) with 13.1% and 12.6% are obtained with the solar cell areas of 6 cm^2^ and 10 cm^2^, respectively, while the flexible module of 54 cm^2^ achieves a PCE of 13.2% [137].

Remarkably, few integrated wearable sensing devices have been reported with both an excellent power supply system and excellent sensing capabilities; therefore, a collaborative effort by researchers from multiple research fields is required.

## 5. Challenges and Perspectives

Recent advances in prototypical wearable biosensors with wide-ranging applications across e-skin, contact lenses, and other types of human organ sensors have accelerated the development of 2D wearable biosensor systems. These devices have a wide range of potential uses in the clinical, consumer, and research sectors. They can offer crucial functionality in the fields of clinical medicine, cosmetics, and digital health that conventional electronic systems cannot. However, there is a long way toward final commercialization, especially in materials quality control and system design. Some concerns and potential technical solutions are provided as follows:

Materials quality-control: First, the efficiency of industrial fabrication of 2D materials is a concern. Commercially, traditional silicon-based electronics and organic semiconductors perform better in terms of production costs and efficiency. Developing innovative methods for the large-scale preparation of high-quality 2D materials with low-cost is extremely urgent. Secondly, due to the good thermal/chemical stability and comprehensive study on the graphene and TMDCs, the majority of the present studies on 2D flexible sensors has concentrated on graphene and TMDCs. Other 2D materials with different energy gaps, such as black phosphorus (BP) and PtSe2, have shown promise for sensing applications. In addition to 2D materials, 2D van der Waals heterostructures have advantages on improving material stability and device performance [138]. Constructing new biosensing devices using such heterostructures has the potential to enhance sensitivity, selectivity, and stability. In addition, the biocompatibility of the synthesized 2D materials and 2D heterostructures should be investigated to enable their practical applications in bioanalysis.

System design: It appears that the system design has faced greater difficulties than the materials side. First and foremost, the system design must incorporate additional utility. Nobody wants to carry about a full-body wearable device. Instead, consumers wish to detect more information with a single device, which necessitates greater work in terms of powered devices and integrated circuit architecture. Furthermore, the dependability and safety of the system are the key concerns. It is crucial to make sure that the materials employed are biocompatible. More importantly, the biosensor system should be sturdy enough to function without heat, leaks, explosions, or other adverse effects. Reliability is a wide concept that encompasses characteristics such as longevity, accuracy, and interference resistance. In the case of glucose sensors, for example, the common method is to modify the electrode with glucose oxidase, which must be preserved in a specific environment and cannot be operated for extended periods. This is not reliable in practical application. To solve these difficulties, researchers must build more studies in device structure design.

Finally, there is the issue of communication and terminals. Since the day the internet age begun, secure and protocol-compatible communication has always been a requirement. The sensing device and the endpoint should preferably communicate wirelessly. Simultaneously, the terminal should preferably be a smartphone or a cloud-based one, all of which contribute to the proliferation of wearable biosensing devices.

Considering all the mentioned challenges, research on a 2D-materials-based wearable biosensor system has only just begun. All of this necessitates a greater understanding and exploration of 2D materials and biosensing, as well as multidisciplinary research collaboration. In this way, the development of 2D wearable sensing technologies may lead to significant advances in life science, thus promoting human health.

## Figures and Tables

**Table 1:** Different materials/techniques for the detection of breast cancer target miRNA.

Materials	Linearity	LOD	Method	Response Time	Target	Ref.
AuNPs/GQDs/GO	1.0 × 10^−15^~1.0 × 10^−9^ M	4 × 10^−17^ M	Electrochemistry	Ultrafast	miRNA-21	[19]
rGo	1.0 × 10^−15^~1.0 × 10^−9^ M	1.0 × 10^−15^ M	FET	Ultrafast	miRNA-21	[20]
MOS_2_	1.0 × 10^−16^~1.0 × 10^−10^ M	3 × 10^−17^ M	FET	Ultrafast	miRNA-155	[21]
M/MoS_2_/Thi/AuNPs/GCE	1.0 × 10^−13^~1.0 × 10^−7^ M	2.6 × 10^−14^ M	Electrochemistry	Ultrafast	miRNA-21	[22]
MOS_2_	1.0 × 10^−15^~1.0 × 10^−10^ M	3 × 10^−16^ M	Electrochemiluminescence	Fast	miRNA-210	[23]
Ag@4-MBA@Au SERS	1.0 × 10^−15^~1.0 × 10^−8^ M	3.98 × 10^−16^ M	Raman spectrum	Common	miRNA-21	[24]
DNA-copper	3.0 × 10^−6^~5.0 × 10^−7^ M	1.7 × 10^−15^ M	Fluorescence	Slow	miRNA-21	[25]
AuNPs/PGEs	2.0 × 10^−10^~3.8 × 10^−7^ M	1.0 × 10^−10^ M	Electrochemistry	Ultrafast	miRNA-21	[26]

**Table 2:** Summary of the four types of e-skin.

	Sensitivity	Response Time	Robust Cyclability	Strain Detection Range	Power Consumption	Cost	Ref.
Resistive	2.14 kPa^−1^	6.7 ms	2000	0.5%~250%	Low	Low	[85]
Capacitive	6.13 kPa^−1^	60 ms	12,000	0%~30%	Low	Low	[86]
Optical	1 mW cm^−2^	Fast	NA	0%~50%	High	High	[90]
Piezoelectric	9.4 × 10^−3^ kPa^−1^	5 ms	NA	NA	0	High	[91]
